# Significant vision improvement and successful prevention of recurrence by electroacupuncture in hemiretinal vein occlusion combined with macular edema: A case report

**DOI:** 10.1097/MD.0000000000028202

**Published:** 2021-12-17

**Authors:** Yan Yan, Yu Chen, ZhiShun Liu

**Affiliations:** a Department of Acupuncture, Guang’anmen Hospital, China Academy of Chinese Medical Sciences, Beijing, China; b New Zealand College of Chinese Medicine, Auckland, New Zealand.

**Keywords:** case report, complementary or alternative therapy, electroacupuncture, macular edema, retinal diseases, retinal vein occlusion

## Abstract

**Rationale:**

Retinal vein occlusion (RVO) is the second commonest retinal vascular pathology, with macular edema (ME) as one of its major complications, which could finally cause vision loss. Anti-vascular endothelial growth factor (anti-VEGF therapy), as the standard therapy, has an unsustainable effect and needs repeated injections, which associates with frequent adverse events and significant economic burden. We reported a patient who had developed RVO and ME, and finally recovered after electroacupuncture treatment.

**Patient concerns:**

A 56-year-old woman complained a decrease of visual acuity in the right eye for 1 year. She received injection of 0.5 mg Conbercept, one of the anti-VEGF therapies, in the vitreous cavity 6 times in 1 year, and finally turned to acupuncture for help out of short-term effectiveness after each injection and high expenditure (CNY 40,800). No other severe medical history was reported.

**Diagnosis:**

Under comprehensive consideration of clinical manifestations and the results of fluorescein fundus angiography and optical coherence tomography, the patient was diagnosed with hemi-RVO and ME.

**Interventions:**

The patient received electroacupuncture 3 sessions per week throughout 8 months (93 sessions in total).

**Outcomes:**

The visual acuity of the patient was improved from 0.6 to 0.9 after the 8-month electroacupuncture treatment and remained stable during the 24-month follow-up; the central retinal thickness remained stable between 350 and 414 throughout the treatment and follow-up periods. Patients regarded the vision-related quality of life as satisfactory. The total expenditure of electroacupuncture treatment was CNY 6045. The patient did not receive any Conbercept injection over the whole period of 32 months. No relevant adverse events occurred.

**Lessons:**

Electroacupuncture might be effective in alleviating the symptoms of hemi-RVO-associated ME, with a potential of long-lasting effect. The frequency of anti-VEGF therapy could be reduced to the most extent, and the possibility of recurrence could be reduced as well, resulting good economic benefits.

## 1 Introduction

Retinal vein occlusion (RVO) is the second most common retinal vascular disorder right next to diabetic retinopathy.^[1]^ It affects more than 16 million people worldwide, mainly those aged between 60 and 70.^[2,3]^ Based on different veins occluded, it can be further divided into branch RVO, central RVO, and hemi-RVO, in which 2 altitudinal quadrants are involved.^[4]^ The vascular pressure increases out of occlusion and may force the fluid and small molecules to leak into surrounding tissues, resulting in local edema eventually.^[5]^ Notably, the complication of macular edema (ME) is the most common cause of visual impairment,^[6]^ which may become irreversible, if not appropriately treated.^[4]^

Anti-vascular endothelial growth factor (anti-VEGF) therapy is recommended as the standard care for ROV-associated ME by guideline.^[4,7]^ It relieves the symptoms via promoting phosphorylation of tight junction proteins, increasing the permeability of vessels, and mediating the breakdown of the blood–retinal barrier.^[8]^ However, anti-VEGF injections are associated with a series of ocular adverse events, such as subconjunctival hemorrhage, PRE tear, mild anterior chamber reaction and hyperemia, and cardiovascular adverse events.^[9]^ In addition, the high expenditure attaches a large economic burden to both individuals and the health care system.^[4]^ Therefore, it is urgent to seek an effective complementary therapy to reduce the usage of anti-VEGF injection and maintain similar efficacy in the meanwhile.

Acupuncture is commonly used to treat intractable eye diseases in clinical practice in China.^[10]^ In this study, we report a patient who developed RVO-associated ME and recovered after electroacupuncture treatment.

## 2 Case presentation

We have obtained written consent from the patient to have the details and images of the case published. Since this is a case report without the patient's privacy and related personal information, the approval of the Institutional Review Board is not necessary.

**Figure 1:**
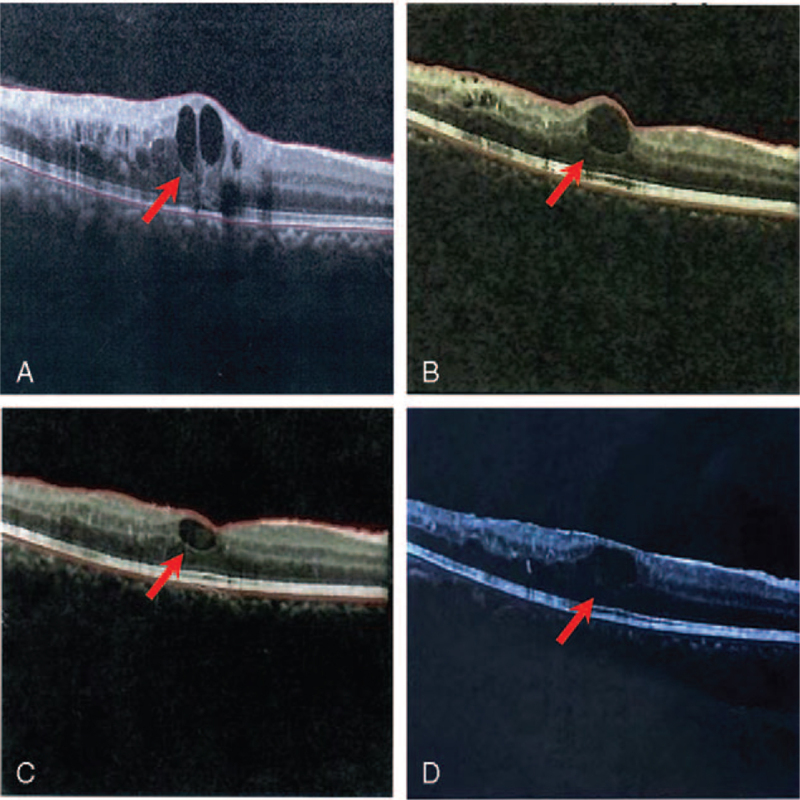
The optical coherence tomography images of the patient's central retinal thickness taken at the first diagnosis (A), before electroacupuncture treatment (B), after 8-month electroacupuncture treatment (C), and after 29-month follow-up (D). The arrowhead is the place with the most severe edema.

A 56-year-old woman without any severe medical history complained visual acuity (VA) worsening in the right eye for 1 year and visited a specialist ophthalmology hospital. The clinical manifestation, fluorescein fundus angiography, and optical coherence tomography suggested a diagnosis of half retinal vein occlusion with ME in the right eye, with a VA of 0.2 and a central retinal thickness (CRT) of 597 μm (Fig. 1A). The patient then received the injection of 0.5 mg Conbercept into the vitreous cavity of her right eye every 4 to 12 weeks, 6 times in total during the following year. The vision of her right eye improved and maintained 1 month or so after each injection. The total expenditure was CNY 40,800. Finally, the patient discontinued the treatment in the ophthalmology hospital, out of the reason of unsustainable effect, injection risk, and heavy cost. Then, she turned to acupuncture for help. The VA of the right eye was 0.6, and the CRT was 398 μm (Fig. 1B) after the sixth injection (before acupuncture treatment).

## 3 Intervention

The patient received electroacupuncture treatment at acupoints of Shangxing (DU23), bilateral Yuyao (EX-HN4), bilateral Sizhukong (SJ23), bilateral Fengchi (GB20), bilateral Taiyang (EX-HN5), and Waiguan (SJ5). Hwato-brand disposable acupuncture needles (size 0.30 × 40 mm), and Hwato-brand SDZ-V electroacupuncture apparatuses were used.

The needles were inserted approximately 10 to 30 mm into bilateral Shangxing (DU23), Yuyao (EX-HN4), and Sizhukong (SJ23) at an angle of 15°; 15 to 20 mm into bilateral Fengchi (GB20) at an angle of 45° toward the other eye; 30 to 35 mm vertically into bilateral Taiyang (EX-HN5); and 15 to 30 mm vertically into bilateral Waiguan (SJ5). After the “de qi” sensation (manifested as numbness, heaviness, distention, and soreness, with a spreading sensation) was achieved, paired electrodes of Hwato-brand SDZ-V electroacupuncture apparatuses were attached to the handle of needles at bilateral SJ23 and GB20. In each session, the electroacupuncture stimulation lasted for 30 minutes in a continuous current waveform, with current frequency at 1 Hz, and current intensity adjusted within the patient's tolerance. The treatment was given 3 sessions per week for 8 months, with a total of 93 sessions (the treatment was ceased in 1 week because the patient had a cold and was unable to visit the hospital). The expenditure of electroacupuncture was CNY 6045.

## 4 Results

At the end of the 8-month electroacupuncture treatment, the VA of the patient was 0.9, and the CRT was 350 μm (Fig. 1C). After the acupuncture treatment was ceased, the patient has been followed up for till month 32. The latest VA examination at month 32 was 0.6 and the latest CRT examination at month 29 was 414 μm (Fig. 1D). No adverse event occurred during the whole period.

The patient had stopped receiving anti-VEGF therapy ever since the beginning of electroacupuncture therapy, for a total of 32 months till now. She perceived a clear vision and a satisfactory vison-related quality of life during both the acupuncture treatment and follow-up periods subjectively. Her recent reexamination in the ophthalmology hospital concluded that the ME had disappeared. Impressed with the effectiveness of acupuncture therapy, the ophthalmologists suggested the patient continue with the acupuncture treatment and receive reexamination as frequently as healthy people.

## 5 Discussion

The patient's VA was improved during the 8-month electroacupuncture treatment, and remained at a steady level during the 2-year follow-up after stopping the treatment; the CRT remained stable throughout the 32-month treatment and follow-up periods. Patients reported that the vision-related quality of life became satisfactory.

The underlying pathogenesis of RVO and associated ME are rather complex and not fully known.^[11]^ The aim of acupuncture therapy is thus to alleviate symptoms and prevent recurrence in the meanwhile.^[8]^ Anti-VEGF shows effectiveness in treating the disease.^[12]^ However, with an intravitreal half-life as short as 2.8 to 6.61 days, it requires repeated intravitreal injections^[13–15]^ frequently and the efficacy might gradually fade away with repeated administration.^[16]^ Additionally, a number of tests are needed to conduct an appropriate treatment regime. Only 2% to 10% patients are reported tolerant to anti-VEGF therapy.^[17–19]^

According to the theory of Traditional Chinese Medicine, RVO-associated ME falls into the category of “sudden blindness”, with “blood stasis” as the major pathology. Acupoints at the local pathology area, together with those points selected as per meridian differentiation theory, are stimulated to dredge the blocked fundus, promote the movement of qi and blood around the fundus and eyeballs, and thus improve the microcirculation around the eyes. Ever since the start of electroacupuncture treatment, the patient has not received any anti-VEGF injection for more than two and a half years, with the result of stable VA and CRT, as well as satisfactory vision-related quality of life. There was no apparent recurrence of the symptoms during the 1 year after the cessation of acupuncture. In light of cost-effectiveness, fewer side effects, and good patient compliance, the therapy of electroacupuncture provides an alternative choice for the patient to treat RVO and ME.

The mechanism behind the effects of acupuncture on RVO is not clear at present. Yan et al^[20]^ reported that acupuncture could inhibit the reactivity of plasma fibrinogen molecular and thus reduce the blood coagulation in rabbits with RVO. It can also reduce the expression of transforming growth and factor β1, inhibit the inflammatory response, and improve the neuroimmune microenvironment.^[21]^ Additionally, electrical stimulation therapy is reported to have the ability to increase retinal blood flow and improve visual function.^[22,23]^

There are certain limitations in this case report. The VA and CRT before the onset of the illness had not been regularly examined, and therefore the relationship between CRT and VA, cannot be fully assessed out of limited data. Since it is just an individual case in clinical practice without a control group, the placebo effects of acupuncture and self-remission of the disease cannot be excluded.

## 6 Conclusion

In summary, the case suggested that electroacupuncture might be effective in relieving the symptoms of hemi-RVO-associated ME. The frequency of positive drug usage has been greatly reduced, and the recurrence of symptoms was prevented over a relatively long term. Meanwhile, good economic benefits and safety have been achieved. It indicates a promising application value although the findings need to be assessed by randomized controlled trials further.

## Acknowledgments

The authors thank the patient for her allowance of the publication of the case report, and for her patience and time during the process. Deep appreciation to the ophthalmologists of Guang’anmen Hospital for their professional guidance.

## Author contributions

**Conceptualization:** Zhishun Liu.

**Data curation:** Yan Yan.

**Investigation:** Yan Yan.

**Methodology:** Zhishun Liu.

**Writing – original draft:** Yan Yan.

**Writing – review & editing:** Yu Chen, Zhishun Liu.
